# Comparative Analysis of Biochemical and Cellular Assay Conditions and the Need for a Buffer That Mimics Cytoplasmic Environments

**DOI:** 10.3390/molecules30173630

**Published:** 2025-09-05

**Authors:** George Kontopidis, Iason-Spyridon Patergiannakis

**Affiliations:** Veterinary School, University of Thessaly, 43100 Karditsa, Greece; ipatergiannakis@vet.uth.gr

**Keywords:** buffer, pH, crowding, cytoplasmic viscosity, PBS, phosphate-buffered saline, ionic strength, in vitro, physicochemical microenvironment, assay conditions

## Abstract

The assessment of a ligand’s activity is typically established by measuring its binding affinity in a biochemical assay, often expressed as K_a_ or K_d_ values. Further validation of its biological activity is achieved through cellular assays. There is frequently an inconsistency between the activity values obtained from those assays, which could delay research progress as well as drug development. Factors such as the permeability, solubility, specificity, and stability of active compounds are usually held responsible for this discrepancy. Even when these values are known, inconsistencies in activity measurements remain challenging to explain. This is not surprising since intracellular physicochemical conditions are undoubtedly different from the simplified conditions used in most in vitro biochemical assays. It is therefore reasonable to assume that these differences would be minimized if biochemical measurements were performed under conditions that more accurately mimic the intracellular environment. These physicochemical conditions can alter the K_d_ values. While the cellular environment has been extensively studied for decades, more recent efforts have focused on obtaining equilibrium and kinetic data directly from in-cell environments. Clarifying molecular crowding, salt composition, and lipophilic parameters inside the cell and thus their effect on molecular equilibrium is a crucial step toward replicating the intracellular environment.

## 1. Introduction

To establish the biological activity of an inhibitor, drug, substrate, natural product, or macromolecule, it is necessary to determine its binding affinity, K_a_ (equilibrium association constant), K_d_ (equilibrium dissociation constant), or IC_50_ (half-maximal inhibitory concentration) or K_i_ (inhibition constant) of the interacting components. These measurements are typically obtained through biochemical assays (BcA) using purified protein targets. K_d_ reflects the affinity between a ligand and its target at equilibrium, defined as

(1) $$K_{d}=\frac{\left[L\right]\left[P\right]}{\left[LP\right]}$$

where [L] is the free ligand, [P] is the free protein, and [LP] is the complex. IC_50_ represents the concentration of an inhibitor required to reduce activity by 50%, and is highly dependent on assay conditions, including enzyme concentrations. However, when determining IC_50_, it is not necessary to know the exact total protein concentration [P]_T_, provided that it remains constant across all assays. K_i_ represents the inhibition constant of an inhibitor for an enzyme and is related to IC_50_ through different equations depending on the type of inhibition. For example, in the case of competitive inhibition, K_i_ is related to IC_50_ via the Cheng-Prusoff equation:

(2) $$K_{i}=\frac{IC_{50}}{\left(1+\frac{\left[S\right]}{K_{m}}\right)}$$

where [S] is the substrate concentration and K_m_ is the Michaelis constant. This relationship highlights that, in competitive inhibition, IC_50_ values depend not only on the intrinsic binding affinity of the inhibitor but also on the substrate concentration.

It is equally important to demonstrate compound activity in cell-based assays (CBAs), which are usually performed in later stages to validate initial screening hits. In many cases, though, cell-based screens are also used in the primary screening phase. When this occurs, it remains necessary to identify the molecular target and develop a bioassay for further validation.

This latter step is vital for establishing a robust structure–activity relationship (SAR) [1]. Notably, IC_50_ values derived from CBAs often differ and may be orders of magnitude higher than those measured in BcAs [2,3,4]. Several factors may account for these discrepancies, including differences in compound solubility, membrane permeability, target specificity, and chemical stability [5,6]. However, even when these parameters are well characterized [7,8], inconsistencies between BcAs and CBAs can remain difficult to explain [9,10,11,12]. We have frequently encountered such discrepancies in our own research [3,13,14,15], a phenomenon also widely reported by other groups [16,17].

This appears to be a persistent issue that has not been effectively addressed, mainly because BcA and CBA teams often generate and report activity data independently. Consequently, these datasets often fail to align within a consistent SAR pattern [3,18]. An increase in BcA activity across a series of compounds does not necessarily correspond to a proportional increase in CBA activity. In many cases, solubility is unlikely to account for this discrepancy, as it often exceeds by orders of magnitude the concentrations used in BcA and CBA [11,12]. In other instances, permeability issues have been proposed as an explanation, even when direct permeability measurements are unavailable [19].

A critical factor is that intracellular physicochemical (PCh) conditions differ markedly from those present in plate wells used for most enzymatic, binding, and routine laboratory bioassays [15,20,21,22,23,24,25]. These differences are expected to affect the diffusion and binding behavior of macromolecules [26], potentially contributing to inconsistencies between BcA and CBA results. Alterations in the PCh properties of assay media can lead to substantial differences in K_d_ values for macromolecular interactions [24,27,28]. The direct measurement of protein-ligand K_d_ values within living cells [11] has offered valuable insights into the role of cytoplasmic crowding in modulating noncovalent interactions. In-cell K_d_ values can differ by up to 20-fold [11,29], or even more [30], from their corresponding BcA values.

These variations in PCh conditions affect not only equilibrium binding but also the kinetics of enzymatic reactions. Experimental data have shown that enzyme kinetics can significantly change [31] (by as much as 2000%) under crowding conditions [32].

Therefore, it is reasonable to assume that such differences could be reduced if BcA measurements were performed under conditions (such as crowding, viscosity, salt, and cosolvent content) that better approximate the intracellular environment. Over the last 40 years, the PCh characteristics of the cytoplasm have been extensively studied [20,21,25,26,33,34,35,36,37]. This body of knowledge could be used to design in vitro solutions that effectively mimic the intracellular environment, an approach that is increasingly recognized and encouraged [38].

Quantifying the differences in PCh parameters between standard BcA conditions and the intracellular environment represents a critical step toward bridging the observed activity gap between BcAs and CBAs, the main objective of this review.

Interestingly, protein crystals may serve as a useful model for the cytoplasmic environment, as they share similar PCh characteristics [33]. Protein crystals typically consist of 30–60% water or solvent by weight, a ratio comparable to the protein-to-water ratio in cytoplasm. At such high concentrations, water exists in two forms; approximately half serves as hydration water, forming strong interactions with protein surfaces. This observation led us to explore the estimation of K_d_ values in crowded environments. We conducted what appears to be the first experiment to estimate K_d_ values using crystallographic data alone [39]. The remaining water in protein crystals behaves as bulk solvent, in terms of its physical and chemical properties [33]. However, properties such as viscosity and salt concentration influence the mobility and behavior of water in this environment, differentiating it from bulk water. We have shown [28] that viscosity, salt concentration, and cosolvent presence (affecting lipophilicity) can significantly influence K_d_ values without denaturing the protein or its interacting ligand (substrate, drug, or inhibitor) [40,41].

In this review, we examine how crowding, salt concentration, and cosolvent content affect equilibrium constants and how these conditions can be modulated in a solution to more accurately simulate the intracellular environment [42,43,44,45,46]. Accordingly, the term “buffering” is used not only to refer to pH and salinity but also to encompass additional parameters, such as macromolecular crowding agents, viscosity-modifying compounds, and cosolvents that modulate solution lipophilicity.

The intracellular and extracellular compartments differ significantly in redox potential, with the cytosol being markedly more reducing due to the presence of protective, reducing agents such as glutathione [47,48,49]. This difference in redox status can influence the oxidation state of protein residues, particularly cysteines and aromatic amino acids, thereby affecting protein folding, stability, and interactions with small molecules [50]. However, the impact of redox potential depends on the specific assay used. To simulate cytosolic redox conditions in biochemical assays, reagents such as dithiothreitol (DTT) or β-mercaptoethanol are sometimes employed. However, their use must be adapted to the specific assay context, as reducing agents may disrupt proteins that rely on disulfide bonds for their structural integrity, leading to protein denaturation [51]. Unlike the other physicochemical parameters discussed in this review, such as crowding, viscosity, salt composition, and cosolvents, which do not affect covalent bonds or compromise the structural integrity of macromolecules and ligands, redox-modifying agents can directly break disulfide bridges. For this reason, the present review does not advocate for the inclusion of a specific redox reagent in cytoplasm-mimicking buffer systems, but acknowledges the importance of redox status as a relevant PCh parameter in cytoplasmic mimicry.

The majority of known drug targets, and nearly all key drug-metabolizing enzymes, are located within the intracellular environment [52]. Nevertheless, the most widely used buffer solution (BS) for studying molecular interactions is phosphate-buffered saline (PBS), which more closely approximates extracellular rather than intracellular conditions. The widespread use of PBS is evident in a simple search on Google Scholar using the keyword “Phosphate Buffer”. This term yields over two million results (out of approximately 400 million total records [53]), whereas the most common protein assay method in biochemistry, “Bradford”, returns slightly fewer than two million results. Although PBS is well-established as a standard for short-term cell maintenance, its suitability for studying binding interactions is questionable. A closer examination of PBS reveals that its dominant cation is Na^+^ (157 mM), with relatively low K^+^ levels (4.5 mM). In contrast, intracellular conditions are characterized by a reverse ratio, with K^+^ concentrations around 140–150 mM and Na^+^ at approximately 14 mM [43,46]. Beyond cation composition, the overall salt concentrations used in PBS do not replicate those found within the cytosol. Although PBS resembles extracellular PCh conditions such as pH, osmotic pressure, and salt type, it fails to capture other critical properties, including viscosity and macromolecular crowding. Common BSs like PBS were designed to maintain cell viability under extracellular-like PCh conditions (pH, osmotic pressure), but they are inadequate for simulating intracellular environments, particularly regarding solute composition, ionic balance, crowding, and lipophilicity [20,21,54]. This discrepancy illustrates the importance of carefully tailoring assay conditions to reflect the physicochemical context of the intracellular space when studying biologically relevant interactions.

## 2. Cytoplasmic Versus Solution Environment

Chemical equilibrium is strongly influenced by the PCh conditions. The dissociation constant (K_d_ = 1/K_a_), which reflects the strength of molecular interactions at equilibrium, is particularly sensitive to parameters such as temperature. In the eukaryotic cytoplasm, temperature is typically maintained at ~37 °C, a condition that is generally replicated in experimental setups. Similarly, pH, another major condition that affects K_d_, fluctuates within a narrow range in the cytoplasm and can be accurately reproduced in vitro using a variety of buffering systems. Despite these similarities, distinct differences in PCh conditions have been observed between physiological and tumor cells.

**Figure 1:**
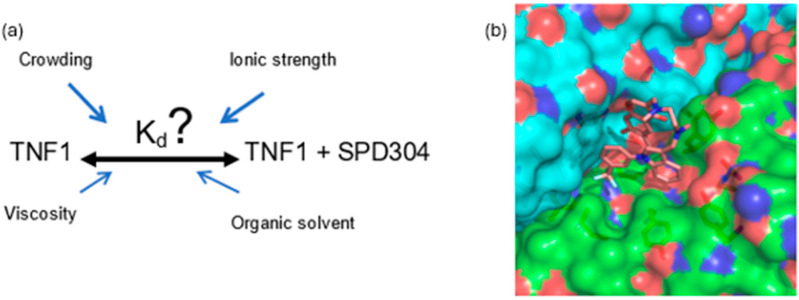
(**a**) Schematic representation of how PhC parameters affect the K_d_ of a protein–ligand complex. (**b**) Structure of the SPD-304 ligand bound to a hydrophobic pocket formed by the TNF1 homodimer interface (PDB ID: 2AZ5). The K_d_ of the TNF1/SPD304 complex can be affected to varying degrees by PCh factors such as macromolecular crowding, viscosity, hydrophobic solvation (organic solvents), and ionic strength (salts) [28]. Image was generated by The PyMOL Molecular Graphics System, Version 1.2r3pre, Schrödinger, LLC.

Other key PCh parameters, including diffusion (affected by macromolecular crowding and viscosity), activity coefficient (modulated by ionic strength), macromolecular conformational dynamics (linked to viscosity), and hydrophobic solvation (influenced by cosolvents), can also significantly impact chemical equilibria. However, these factors are frequently overlooked in standard in vitro assays [26,36,37,55]. In this review, we systematically examine each of these parameters and their relevance to cytoplasmic mimicry (Figure 1).

## 3. Intracellular pH and Differences with Extracellular pH

The cytoplasmic concentration of H^+^ ions is one of the most fundamental and extensively studied parameters in cell biology, and influences many biological processes [45,56,57,58]. Protein-ligand interactions are often influenced by pH-dependent changes in the protonation states of ionizable groups on either the ligand or the protein. Binding itself can induce such protonation shifts and alter pK_a_ values due to factors such as desolvation, electrostatic interactions, or conformational changes upon complex formation [59]. These effects must be carefully considered when interpreting activity measurements and are particularly relevant in the context of this review.

In eukaryotic cells, intracellular pH (pH_i_) varies depending on the cell type, developmental stage, and metabolic state, typically ranging from 6.7 to 7.7 [42,60]. In mammalian cells, cytoplasmic pH values are commonly reported around 7.2–7.4 [56,58], with a median value of approximately 7.3 often considered physiologic [61].

Intracellular pH regulation is a complex process involving proton-pumping ATPases, Na^+^/H^+^ exchangers (NHEs), and bicarbonate transporters, all of which contribute to maintaining acid-base balance [56,62]. Proton gradients and metabolic processes, particularly those that generate acidic byproducts, can lead to cytoplasmic acidification, which is counteracted by these regulatory mechanisms [56]. The negative intracellular membrane potential further facilitates H^+^ influx and the efflux of negatively charged bases, contributing to the fine-tuning of pH_i_ [56,63]. Interestingly, even in the acidic microenvironment of tumors, cancer cells have a slightly more alkaline cytosol compared to normal cells, with pH_i_ reaching 7.6–7.7 [61,63,64,65]. Furthermore, different cell lines exhibit distinct intracellular pH ranges. For example, fibroblast cell lines typically maintain pH_i_ of approximately 7.4–7.7, whereas transformed or cancerous cell lines tend to have slightly lower values, ranging from 7.0 to 7.4 (Table 1) [66].

Because the nucleus contains large pores permeable to small molecules, early studies hypothesized that nuclear pH is identical to cytosolic pH due to unrestricted proton exchange [56]. However, more recent studies have challenged this assumption, suggesting that the nuclear environment may possess distinct pH regulatory mechanisms [67,68,69]. For example, in HeLa cells, nanowire waveguide-based pH measurements revealed a nuclear pH of approximately 6.9, compared to a cytosolic pH of 7.1 [68]. Another study using a colorimetric imaging method in L929 cells reported an even lower nuclear pH of ~5.6, whereas the cytoplasmic pH remained at ~7.1 [69]. These findings imply that nuclear pH may be influenced by intracellular compartmentalization, active proton transport and nuclear-specific buffering systems. Different cellular organelles have markedly different pH values that are critical to their specific functions. For instance, lysosomes have an acidic environment that can reach pH ~4.5, while mitochondria maintain an alkaline matrix of ~8.0 [56]. However, the detailed mechanisms by which the pH levels are controlled are beyond the scope of this review.

Maintaining extracellular pH (pH_e_) is equally crucial. Under normal physiological conditions, pH_e_ is slightly alkaline, usually ranging between 7.3 and 7.4. In contrast, the tumor microenvironment often exhibits a more acidic pH_e_, which can drop below 6.7, depending on tumor aggressiveness and metabolic activity [61]. In addition to tumors, several pathological conditions, including inflammation, ischemia, respiratory disturbances, and metabolic disorders, can significantly alter interstitial pH. Moreover, transient pH_e_ flunctuations can occur during normal physiologic events, such as in skeletal muscle during intense exercise, where lactic acid accumulation leads to temporary acidification of the local extracellular environment [63].

**Table 1:** Intracellular pH in different types of cells.

Cell Types	pH_i_
Physiologic mammalian cells	7.2–7.4 [62]
Tumor cells	~7.1–7.7 [65,66]
Fibroblast cell line	7.4–7.7 [67]
Transformed cell line	Typically, 7.0–7.4 [67]
	L929: ~7.1 [69]
	HeLa ~7.1 [68]

## 4. Molecular Crowding

Molecular crowding represents one of the most pronounced physicochemical differences between the BcA environment and the cytoplasm. Several studies have noted that the crowded intracellular environment is one of the most important factors affecting macromolecular interactions within the cell [25,26,27,33,35,37,70,71].

Within the cell, the cytoplasm is predominantly composed of water, which accounts for approximately 70% of its total mass [72]. The remaining dry mass consists largely of proteins, with macromolecules collectively accounting for 10–40% of the total cellular volume [72,73]. These macromolecules form a dense, porous, viscoelastic meshwork that restricts the diffusion of larger particles and contributes to the physical complexity of the intracellular environment [73]. In addition to the macromolecular network, the cytoplasmic environment imposes diffusion limits on macromolecules due to its crowded and mesh-like organization, which further constrains molecular mobility and accessibility [74].

Eukaryotic cells contain numerous intracellular structures, including ribosomes, proteasomes, transport vesicles, stress granules, the endoplasmic reticulum, the Golgi apparatus, mitochondria, peroxisomes, lysosomes, and endosomes, that influence the cytoplasm. These components contribute to intracellular crowding either as mobile macromolecular solutes or as spatially constrained obstacles, such as cytoskeletal filaments and larger vesicular structures [54]. Mobile structures behave like solute particles and affect diffusion and reaction equilibria, whereas larger organelles, which contain distinct internal environments, act as volume-excluding barriers [54]. Cytoskeletal filaments, although composed of proteins, serve as physical obstacles because of their extended length and anchoring within the cell, forming part of a cytoplasmic meshwork that restricts molecular mobility [75,76]. Larger organelles and vesicular compartments function more as volume-excluding boundaries than as dynamic solutes. These membrane-bound structures maintain distinct internal PCh conditions, such as the acidic pH found in lysosomes and endosomes or the redox differences observed in mitochondria and peroxisomes, which further differentiate them from the surrounding cytosol [77,78,79]. While such compartmentalization is difficult to replicate in vitro, it has critical implications for macromolecular diffusion, crowding, drug distribution, and binding equilibria. These factors must be carefully considered when interpreting biochemical data derived from simplified assay systems. For example, vesicular compartments such as lysosomes and endosomes maintain an acidic pH, which can affect the protonation state and intracellular accumulation of weakly basic drugs [77,78].

It is important to note that crowding and viscosity are closely related but fundamentally distinct concepts. A viscous solution can be created using a concentrated sucrose solution; however, such a solution is not considered crowded. It represents a homogenous medium in which small sucrose molecules can make attractive interactions with neighboring sucrose molecules. In contrast, molecular crowding arises primarily from the presence of macromolecules that behave as sterically excluded hard spheres, each occupying a volume from which other molecules are excluded. This distinction becomes particularly evident when comparing solutions of the polysaccharide Ficoll (~400,000 Da) and disaccharide sucrose (342 Da) at the same concentrations. At 5% *w*/*v*, both Ficoll and sucrose solutions exhibit similar density values (~1.03 g/mL). However, their physicochemical properties differ significantly: the osmolality of the sucrose solution is approximately 150 mOsm/kg H_2_O, while that of Ficoll is around 2.5 mOsm/kg H_2_O. Similarly, the intrinsic viscosity of a 30% *w*/*v* sucrose solution is approximately 2.3 (cP), compared to 75 cP for a 30% *w*/*v* Ficoll solution [80,81]. These examples underscore the importance of selecting appropriate crowding agents when designing experiments intended to replicate intracellular crowding conditions.

These differences have been clearly analyzed at the microscopic level by Ernst, D. et al. [82], who tracked the diffusion trajectories of a 50 m particle in both viscous and crowded solutions. The viscous solution consisted of 60% *w*/*w* sucrose, while the crowded solution was composed of 30% *w*/*w* of the polysaccharide Dextran500, half the mass concentration but with significantly greater molecular size. The mean square displacement (MSD) of the diffusing particle was evaluated using time-dependent diffusion coefficients, D(t). In the sucrose solution, D(t) remained constant over time. In contrast, the Dextran500 solution exhibited subdiffusive behavior, with D(t) = 1/t^0.2^. The two systems also exhibit significantly different gyration ellipsoids, reflecting distinct asphericity in the particle’s random walk.

Simulated fluids composed of 7.5% sucrose and 19% polyethylene glycol 10,000 (PEG 10,000), or 10% sucrose and 15% PEG 10,000, have been shown to closely replicate not only the viscosity but also the T_2_ relaxation time and diffusion coefficient of *E. coli* cytoplasmic fluid containing ~100 mg/mL protein, thereby providing a reliable experimental model of its crowded, viscoelastic environment [83].

In general, crowding restricts the movement and conformational freedom of biomolecules, a phenomenon known as the excluded volume effect, which effectively reduces the system’s entropy (S) [84]. Importantly, this reduction in entropy does not necessarily cause a change in the overall free energy, as the Gibbs free energy (ΔG = ΔH − TΔS) depends on the difference in entropy between products and reactants.

In the cellular environment, where macromolecules such as proteins and nucleic acids reach concentrations exceeding 350 mg/mL, crowding effects become particularly relevant [85]. To simulate these effects in vitro, crowding agents such as PEG, Ficoll, and dextran are widely employed to increase both viscosity and steric hindrance [86]. Under these conditions, changes in the solution, even without altering the concentration of reactants or products, can affect ΔG°, which is directly related to K_a_ through the equation

ΔG°, = 2.303 × R × T × pK_a_ (3)

Macromolecular crowding alters RNA folding by restricting conformational space and destabilizing the unfolded state entropically, shifting ΔG°, without changing the reactant concentration [85].

Similar conformational responses to crowding have been observed in proteins. Intrinsically disordered proteins (IDPs), which lack stable tertiary structure under physiological conditions, may transition toward more compact ensembles when exposed to molecular crowding [86,87]. The C-terminal domain of histone H1, which is intrinsically disordered in dilute solution, adopts a more compact structural ensemble in the presence of crowding agents such as Ficoll 70 or PEG 6000, indicating a native-like secondary structure and compaction [87].

However, IDPs do not respond uniformly to crowded environments [86]. In the case of the bacterial transcriptional regulator CytR, stabilization arises primarily from PEG-induced steric hindrance and reduced configurational entropy, representing a dominant entropic mechanism. Crowding agents act as inert, noninteracting spheres that constrain the conformational freedom of unfolded protein chains [86].

## 5. Intracellular Salt Differences with Respect to Extracellular pH and Common Buffers

Ionic strength is determined by the concentration of various ionic species, including both cations and anions, and is a major factor influencing osmolarity. It significantly affects electrostatic interactions, impacting enzyme activity, molecular structure, gene expression, particularly of osmolarity-sensitive genes, the diffusion of biomolecules on membranes, and other cellular functions [88].

Ions play a central role in shaping the PCh environment of the cell. For example, potassium (K^+^) is the primary intracellular osmolyte, and its levels per cellular protein content correlate strongly with intracellular water content [43].

The aim of this review is not to provide an exhaustive analysis of all anions and cations present in the cytoplasm. Instead, it focuses on identifying those with the highest intracellular concentrations and, therefore, those most relevant for modulating PCh behavior. While it is theoretically possible to replicate the cytoplasmic composition by quantifying the concentrations of dozens of ions and small organic molecules, such an approach is experimentally complex and does not yield a practical or standardized buffer system. Therefore, the key goal of this review is to clarify which components critically influence binding reactions and to propose solution conditions that more accurately reflect the intracellular environment in a standardized and accessible manner.

### 5.1. Cations

Metal ions are important for many physiological processes. Certain metals, such as sodium (Na), potassium (K), magnesium (Mg), calcium (Ca), manganese (Mn), ferrum (Fe), cobalt (Co), zinc (Zn), copper (Cu), and molybdenum (Mo), are considered essential trace elements [89,90]. Nickel (Ni) has been proposed as a potentially essential trace metal [89,90], yet its status in higher organisms remains debated. Although it is recognized as essential in certain prokaryotes and lower eukaryotes, no specific biochemical function has been conclusively identified in mammals. Furthermore, the absence of nickel does not disrupt the life cycle of higher organisms [91], and it is typically present at very low intracellular concentrations [92]. Different species may require additional trace metals such as vanadium (V) and tungsten (W) [89].

Cells regulate and buffer intracellular metal ion concentrations in inverse correlation with the Irving–Williams series. As a result, metals forming the most stable complexes, such as Cu^+^ and Zn^2+^, are maintained at extremely low free concentrations. Buffered concentration of Cu^+^ is predicted to range from zeptomolar to femtomolar, whereas Zn^2+^ is typically regulated within a few orders of magnitude around the picomolar scale [93]. Even though some of these (e.g., Zn) exist in trace amounts relative to total body mass, they are essential for a wide array of biochemical processes.

Depending on their size and concentration, ions may cross membranes via diffusion (facilitated or not) or through active transportation [90]. Their PCh properties directly influence their biological behavior. Metal ions with weak to moderate ligand interactions tend to exhibit higher mobility across cellular compartments. In contrast, Zn and transition metals, including Fe, Cu, Co, Mn, and Mo, often form stable, kinetically inert complexes, limiting their intracellular mobility [89]. Metal ions are abundant in both intracellular and extracellular fluids, where they interact with charged and polar groups of biopolymers exposed to aqueous environments. The PCh properties of these cations, such as their ionic radii, can vary significantly depending on both the specific ion and its oxidation state; for example, Mn^+4^ has an ionic radius of 0.52 Å, whereas K^+^ has an ionic radius of 1.33 Å [94]. Among the most abundant intracellular ions are Mg^2+^, Na^+^, and K^+^ with ionic radii of 0.66, 0.95, and 1.33 Å, respectively. Moreover, the hydration shell structure and geometry surrounding each ion are highly ion-specific, affecting their mobility, reactivity, and interaction with biomolecules [94].

Sodium is primarily found in the extracellular fluid, where its concentration ranges between 135 and 145 mM, more than tenfold higher than the typical intracellular concentration of approximately 10 mM [95,96]. Sodium is the principal determinant of plasma osmolality [97]. In contrast, potassium is the dominant intracellular cation. Most of the total potassium in the body is found in the intracellular fluid at concentrations typically ranging between ~140 and 150 mM. The extracellular concentration of potassium is maintained at ~3.5–5 mM [98].

Magnesium is the second most abundant intracellular cation after potassium. In mammalian cells, total intracellular Mg^2+^ concentrations typically range from 17 to 20 mM, but only a small fraction, approximately 0.5–1 mM, exists in the free, ionized form (Mg^2+^). In extracellular fluids, magnesium concentrations are generally maintained between 1.2 and 1.4 mM, with approximately one-third bound to biomolecules [99]. Other essential cations are found in significantly lower concentrations [89,90,94,100,101,102,103,104,105], as shown in Table 2.

### 5.2. Anions

Several key anionic species are present within mammalian cells, including chloride (Cl^−^), bicarbonate (HCO_3_^−^), inorganic phosphates (P_i_), sulfate (SO_4_^2−^), and negatively charged macromolecules such as proteins, which typically carry a net negative charge at physiological pH. The inorganic anions differ in ionic radii, a property that influences their mobility, hydration shells, and interactions with macromolecules. Among them, SO_4_^2−^ and the physiologically relevant protonated forms of phosphate (H_2_PO_4_^−^) and bicarbonate (HCO_3_^−^) have relatively large ionic radii at 2.18, 2.13, and 2.07 Å, respectively, while Cl^−^ has the smallest ionic radius at 1.81 Å [107].

In the extracellular compartment, P_i_ concentrations range from 2.87 to 4.81 mM in serum, which is nearly 100-fold higher than the concentrations typically found within cells. Phosphate exists in multiple states: bound to proteins, complexed with cations such as Ca^2+^, Mg^2+^, and Na^+,^ and as free inorganic phosphate in equilibrium between HPO_4_^2−^ and H_2_PO_4_^−^. At physiological pH (~7.4), the ratio of HPO_4_^2−^:H_2_PO_4_^−^ is approximately 4:1 [108].

Chloride is the predominant extracellular anion and plays a central role in regulating membrane potential, modulating ion transport, and influencing intracellular pH. Intracellular Cl^−^ concentration varies significantly across cell types, typically ranging from 5 to 60 mM [109,110]. Extracellular Cl^−^ levels are tightly regulated, typically maintained between 95 and 120 mM across species, with a narrower range of 97–107 mM observed in humans [110].

Cytosolic HCO_3_^−^, typically maintained at ~12 mM, is crucial for intracellular pH regulation. Its homeostasis is tightly linked to extracellular HCO_3_^−^ levels, which range from 25 to 29 mM. Bicarbonate transport across membranes contributes significantly to pH buffering and acid-base balance within the cell [56,95].

Plasma sulfate concentrations are species—dependent; in humans, SO_4_^2−^ levels range from 250 to 300 μM [111]. In addition to inorganic anions, negatively charged proteins constitute a major component of the intracellular anion pool, with concentrations in mammalian cells averaging ~138 mM, while their concentration in blood plasma is approximately 9 mM [95]. These ions possess different PCh properties, such as hydrodynamic radius, as presented by Kadhim and Gamaj [107].

Common buffers that are used in biochemical assays typically contain a limited selection of ions, often restricted to those necessary for maintaining pH stability. These typically include monovalent (Na^+^, K^+^, Cl^−^) and occasionally divalent ions (Mg^2+^), but lack the ionic complexity observed in physiological fluids. Specifically, 1X PBS, with an ionic strength of 0.156 M, contains NaCl (137 mM), KCl (2.7 mM), Na_2_HPO_4_ (10 mM), and KH_2_PO_4_ (1.8 mM); CaCl_2_ (1 mM), and MgCl_2_ (0.5 mM) may also be added [112,113]. The resulting ion composition includes Na^+^, K^+^, Cl^−^, HPO_4_^2−^, H_2_PO_4_^−^, Ca^2+^, and Mg^2+^. At pH 7.4 the relative concentrations of HPO_4_^2−^ and H_2_PO_4_^−^ can be determined using the Henderson–Hasselbalch equation: pH = pKa + log ([HPO_4_^2−^]/[H_2_PO_4_^−^]). Final ion concentrations are summarized in Table 3.

HEPES is a widely used organic zwitterionic buffering agent with a pKa of 7.55, making it highly effective for maintaining pH near physiological levels. It has a topologically polar surface area (TPSA) of approximately 89.5 Å^2^ [114], which contributes to its high solubility and minimal membrane permeability. Standard HEPES buffer is prepared using HEPES, dissolved in deionized water (dH_2_O) with NaOH added to adjust the pH. HEPES is frequently used to formulate an extracellular saline buffer for in vitro studies. A typical composition of such a buffer (pH 7.3) includes the following final concentrations: 140 mM NaCl, 5.4 mM KCl, 2 mM CaCl_2_, 10 mM MgSO_4_, 5 mM HEPES, and 10 mM glucose.

Moreover, HEPES is used to prepare Hank’s buffer with HEPES (HHBS), which is widely used in cell culture applications. HHBS comprises 1.26 mM CaCl_2_, 0.49 mM MgCl_2_, 0.41 mM MgSO_4_, 5.33 mM KCl, 0.44 mM KH_2_PO_4_, 138 mM NaCl, 0.34 mM Na_2_HPO_4_, 4.17 mM NaHCO_3_, 5.56 mM d-glucose, and 20 mM HEPES [115,116]. The final ionic concentrations of the extracellular saline buffer and HHBS are provided in Table 4.

Tris buffer is commonly prepared using 1 M tricine as the primary buffering agent, with the pH subsequently adjusted to the desired range. Tris has a pKa of approximately 8.1 at 25 °C, which provides effective buffering capacity in the pH range of 7.0 to 9.0 [117]. It also has a TPSA of 86.7 Å^2^, which contributes to its high aqueous solubility and minimal membrane permeability [118]. This buffer system is widely used in electrophoresis applications, particularly for protein separation, due to its effective buffering capacity across a broad pH range [119]. Tris-buffered saline (TBS) is frequently used for washing cell cultures and maintaining isotonic conditions during experimental procedures. TBS is typically adjusted to pH 7.4 and contains 99.88 mM Tris, 136.8 mM NaCl, and 2.68 mM KCl. The final ionic concentrations in TBS are as follows: Na^+^ 136.80 mM, K^+^ 2.68 mM, and Cl^−^ 139.48 mM [120].

## 6. Intracellular Lipophilicity (Hydrophobic Effect) and Differences from Common Biochemical Buffers (BPS Buffer)

Up to 30% of the total weight of the cytoplasm is composed of organic compounds [21]. Thus, a significant portion of the cytoplasmic mass is not strictly hydrophilic. Hydrophobicity refers to a compound’s aversion to water, whereas lipophilicity describes its affinity for lipid environments and governs solute-solvent interactions. Consequently, lipophilicity plays a central role in determining the partitioning behavior of molecules across biomembranes and other lipophilic compartments [121].

Lipophilicity is quantitatively expressed by the partition coefficient (logP), which reflects a compound’s distribution between a nonpolar and an aqueous phase. For ionizable compounds, the distribution coefficient (logD) is preferred, as it accounts for the compound’s ionization state at a specific pH, and therefore provides a more accurate representation of its behavior under physiological conditions [122]. It is a key PCh property that describes the balance between hydrophobic (nonpolar) and polar interactions, calculated using the formula [123]:

(4) P=Drug moleculeoctanolDrug moleculewater

A LogP range of 1–4 typically provides an optimal balance between sufficient membrane permeability and adequate aqueous solubility [124]. These observations indicate that the cytoplasm does not behave like pure water or pure octanol, but rather exhibits intermediate, amphiphilic characteristics.

Unfortunately, there is no straightforward method for defining the lipophilicity of a complex mixture such as the cytoplasm. However, it is reasonable to consider the intracellular environment as amphiphilic. Lipophilicity plays a crucial role in predicting absorption, transport, and distribution in biological systems. A central force underpinning these interactions is the hydrophobic effect, which drives nonpolar molecules to minimize their exposure to water, thereby influencing many cellular biochemical processes. This effect can be modulated by cosolvents, such as alcohols, denaturants, or osmolytes, which alter the structure and interactions of the solvent [55]. Experimental studies have demonstrated that changes in cosolvent concentration, such as ethanol, can affect

ΔG° (ΔG° = ΔH° − T ΔS°)(5)

thereby altering either ΔH°, ΔS°, or both.

Most biochemical experiments (including binding and enzymatic assays) are performed in aqueous solutions. In such contexts, cosolvents are generally added to enhance solubility rather than to mimic the cytoplasmic environment. However, it is well established that solution lipophilicity influences binding affinity via the hydrophobic effect [41,55]. Notably, the most commonly used buffer, PBS, does not contain a cosolvent to increase lipophilicity. A buffer solution containing an amphiphilic compound such as HEPES more closely resembled the cytoplasmic environment.

To effectively navigate the complex cellular environment, a molecule must exhibit a balance between hydrophilicity and lipophilicity. It must be hydrophilic enough to remain soluble in aqueous environments (extracellular fluid and cytosol), yet sufficiently lipophilic to cross the hydrophobic core of cellular membranes. Excessive lipophilicity, however, may lead to sequestration within the interior of the membrane [122]. Moreover, even minor pH differences across cellular compartments can profoundly affect the PCh behavior of weak acids and bases, particularly their ionization state, solubility, membrane permeability, and distribution [125].

Despite these complexities, commonly used buffers such as PBS, Tris buffer, and HEPES buffer are aqueous-based solutions. These buffers are typically optimized for chemical stability and pH control, but they fall short in replicating key biological parameters such as ionic strength, macromolecular crowding, redox potential, and the dynamic composition of ions and small solutes present in living systems.

## 7. Proof of Concept: Alignment Between In Vitro and Cellular Assays

Affinity values determined under dilute aqueous conditions may significantly overestimate binding strength compared to measurements obtained within the cellular environment. In-cell assessments of the spliceosomal protein U1A and its RNA partner, stem loop 2 (SL2), in U-2 OS cells revealed that the apparent binding affinity was reduced by up to two orders of magnitude relative to in vitro measurements. This discrepancy was attributed to the highly interactive intracellular environment, where weak nonspecific interactions compete with specific binding events, effectively diminishing complex stability [126].

Recent studies have validated the concept that cytoplasm-mimicking buffer formulations can significantly improve the alignment between in vitro and cellular observations. For example, Davis et al. demonstrated that a buffer containing 150 mg/mL Ficoll combined with 60% lysis buffer effectively reproduced the intracellular stability patterns of phosphoglycerate kinase (PGK) and the variable major protein-like sequence expressed (VlsE). This formulation was proposed as a practical in vitro model to simulate cytoplasmic crowding for studies of protein interactions [127].

Similarly, Knab et al. reported that the stabilization of a λ_6–85_ protein fragment observed in live cells could not be replicated using Ficoll alone, indicating that steric crowding alone was not sufficient. Instead, stabilization was reproduced using a mixture containing mammalian protein extraction reagent (M-PER™), which is capable of engaging in chemical interactions. They proposed a cytomimetic solution consisting of 150 mg/mL Ficoll and approximately 20% *v*/*v* M-PER™ as a more effective medium for mimicking the intracellular environment in studies involving peptides, RNA, and proteins [128]. A combination of 20% *v*/*v* M-PER™ and 150 g/L PEG 10,000 was found to closely mimic the cytoplasmic environment in terms of its effects on the folding of the low-melting (G12A-C23U) variant of the 4U RNA (lm-4U RNA), accurately reproducing the stabilization and destabilization observed in living cells as a result of macromolecular crowding and surface interactions [129].

Notably, these formulations commonly include a macromolecular crowding agent such as Ficoll in combination with a chemically interactive component, often a detergent or amphiphilic reagent, underscoring the necessity of simultaneously addressing both steric and lipophilic aspects of the intracellular environment. This pattern supports the rationale for incorporating both crowding agents and mild lipophilic cosolvents, such as DMSO, into cytomimetic buffer systems to more faithfully replicate cellular physicochemical conditions.

## 8. Key Thoughts

This review highlights the significant discrepancies between the PCh properties of the cytoplasm and the simplified conditions typically employed in BcAs. The cytoplasm is a densely crowded, ionically heterogeneous, and dynamically regulated environment. It contains high concentrations of macromolecules, distinctive ionic ratios, and tightly controlled pH buffering systems, all of which differ markedly from the static and dilute conditions used in most BcAs.

Luchinat and Banci (2022) underscored this gap between BcAs and CBAs [130]. In light of this, one might question the necessity of buffer development when modern techniques allow direct measurement of equilibria and dissociation constants within cells. Although in-cell techniques can bridge this gap, they are time-consuming, require expensive equipment such as nuclear magnetic resonance (NMR), and demand technical expertise. Cellular thermal shift assay (CETSA) allows the evaluation of ligand binding in live cells. CETSA can be used to determine *IC*_50_ values for individual protein targets without the need for intracellular-mimicking buffers [131]. However, CETSA measurements are typically performed at elevated temperatures, higher than the physiological 37 °C, which poses a limitation when attempting to determine biologically relevant K_d_ values [30,132].

As an alternative, we propose the development of cytoplasm-mimicking buffers that parameterize key PCh features and are compatible with standard biochemical techniques. Such buffers would enable more physiologically relevant studies while remaining cost-effective and experimentally accessible.

The widespread use of PBS and similar aqueous buffers in BcA, while convenient and standardized, raises concerns regarding the accuracy with which such conditions mimic the intracellular environment. The widespread use of phosphate-buffered saline (PBS) in BcA is primarily due to its simplicity, reproducibility, and low cost. Its buffering capacity arises from phosphoric acid, a triprotic acid with pKa values of ~2.1, ~7.2, and ~12.4, enabling it to maintain various pH levels [113]. Additionally, phosphate ions are natural constituents of biological systems, and PBS is typically used at pH ~7.4 to mimic the physiologic extracellular pH [92,133]. However, it lacks key intracellular features, such as high macromolecular crowding, lipophilicity, and pH regulation under CO_2_-enriched conditions. Moreover, PBS can affect enzymatic activity, induce secondary precipitation (e.g., in the presence of ethanol), and interfere with enzyme kinetics, protein dynamics, and structural techniques such as X-ray crystallography [92]. Furthermore, sodium is the dominant cation in PBS, whereas potassium predominates in the cytoplasm. Differences in ionic composition can significantly affect protein-ligand interactions and binding affinity measurements [28,134]. Additionally, PBS contains no organic or crowding agents and, therefore, fails to replicate cytoplasmic viscosity and excluded volume effects.

In cases where PBS is unsuitable, alternative buffers have been proposed [92]. HEPES, a widely used zwitterionic buffer, offers a useful pH range of 6.8–8.2 [116,135]. and is well-suited for cell culture under CO_2_-enriched conditions due to its stable buffering capacity [136]. Nevertheless, HEPES has some limitations considering the modification of organic molecules [116,137] and the replication of intracellular features such as macromolecular crowding or lipophilic partitioning. Another alternative is Tris, which has a pKa ~8.1, resulting in an effective buffering range between ~7–9, but loses buffering capacity below pH 7.4, and is sensitive to temperature changes [117]. Phosphate buffers (Na_2_HPO_4_–NaH_2_PO_4_) offer a buffering range of pH 5.8–8.0 and can be useful in slightly acidic solutions. Although cytoplasmic pH is typically ~7.3, this can vary depending on cell type and physiological or pathological conditions. Thus, buffer pH should be tailored to the cellular model used.

Importantly, effective buffer design must also account for ion composition, salinity, and ionic strength. For example, intracellular concentrations of Na^+^, K^+^, Cl^−^, Mg^2+^, and other ions should be reflected in the solution to preserve biological relevance [43,138]. In practice, adjusting pH with NaOH or HCl often introduces excess salts (e.g., Na^+^, Cl^−^), whereas phosphate-based systems allow pH modulation by adjusting ratios of NaH_2_PO_4_ and KH_2_PO_4_, avoiding ionic overload. Neglecting such interdependencies can compromise experimental outcomes, especially in pharmacokinetics, enzyme kinetics, and protein folding studies, where the PCh context is critical.

The use of cosolvents is also important. DMSO, for instance, is often added to improve solubility, but it can affect protein structure and function by altering the dielectric constant of the medium [139]. Although low concentrations of DMSO (typically below 10%) are generally considered non-disruptive [41,140], certain proteins may still be sensitive, with risks of destabilization, degradation, or aggregation [141].

These examples reflect a broader issue in experimental biology, which is the standardization of methods at the cost of physiological relevance. Despite growing evidence of the limitations of traditional buffer systems, their widespread use delays the adoption of more accurate models. A shift toward evidence-based, cytoplasm-mimicking buffers would better reflect intracellular conditions and enhance the biological validity of biochemical studies.

By integrating key parameters such as macromolecular crowding agents, physiologic ion ratios, appropriate pH ranges, and low levels of cosolvents that mimic cytoplasmic amphiphilicity, the quality and translational potential of in vitro assays can be significantly improved. This approach would benefit a wide range of fields, including enzymology, biochemistry, molecular biology, X-ray macromolecular crystallography, and drug discovery.

### Applications and Implementations

The broader implications of adjusting PCh assay parameters extend across several disciplines in biomedical research and molecular sciences. Improving buffer realism directly improves data accuracy and reproducibility in various high-impact fields.

Molecular Biology: Although the current review focuses on cytoplasmic conditions, the nuclear environment shares many similarities, particularly in terms of crowding conditions and ionic strength [56]. Thus, the conclusions regarding the influence of these parameters on K_d_ also apply to the nucleoplasm. Fundamental molecular mechanisms, such as DNA-binding and protein interactions, are affected by salt concentration and crowding [70].

Crystallography: A long-standing debate persists regarding the biological relevance of crystallized proteins compared to their dynamic intracellular counterparts. Interestingly, crystallization media share several key characteristics with the cytoplasm. One major similarity is the water content (~70% w/v) found in both the cytoplasmic environment and in the crystallographic mother liquor used to grow protein crystals. Moreover, the high-salt and high-viscosity conditions of crystallization buffers do not typically denature the target protein. However, soaking experiments are often unsuccessful [142], and the ligand does not bind to the protein [143], leading to wasted time and resources. Incorporating more realistic buffer environments could improve ligand-protein interactions during crystallographic workflows.

Enzymology: K_d_ and K_m_ are closely related, and under rapid equilibrium conditions, the two values are theoretically identical [144]. Any inaccuracies in K_d_ measurement caused by non-physiological buffer conditions can distort enzyme kinetic interpretations. It has been demonstrated that enzymatic reactions, such as the phosphorylation pattern of mitogen-activated protein kinase (MAPK), are significantly affected by cytoplasmic crowding [145], as well as by other PCh parameters, such as ionic strength and lipophilicity, both of which are affected by cosolvent concentrations [146].

Drug Discovery: Intracellular drug targets are estimated to represent almost 50% of all protein targets [147]. A major challenge in drug discovery lies in the discrepancy between compound activity observed in BcAs and that observed in cellular, tissue or in vivo models [38]. Even when the exact intracellular concentration of the active compound is known, there are significant differences between BcAs and CBAs potencies [9]. This discrepancy further underscores the extent to which these environments differ, as reflected in the variability of compound activity values.

## 9. Conclusions

This review highlights the need to reevaluate the use of simplified aqueous buffers such as PBS in vitro biochemical studies. While these systems offer convenience and standardization, they fail to replicate the complex and tightly regulated PCh environment of the cytoplasm. Critical parameters such as pH, ionic composition, macromolecular crowding, and viscosity significantly influence protein folding, ligand binding, and enzymatic activity, yet they are often overlooked in standard buffer systems. To enhance the biological relevance and predictive power of BcAs, buffer formulations should aim to more closely approximate cytoplasmic conditions. Limitations in buffer design include the inherent difficulty of replicating the full complexity of the intracellular environment, as not all ionic species and physicochemical parameters can be precisely reproduced or standardized. Consequently, certain factors, such as specific trace ions or cytoplasmic viscosity, may need to be deprioritized or excluded depending on the experimental objective.

Based on the analysis presented here, we propose a set of physicochemically relevant parameters and components to guide the formulation of a cytoplasm-mimicking buffer system. This formulation aims to strike a balance between physiological relevance, experimental feasibility, and ease of standardization (Table 5). While the proposed buffer composition remains theoretical, it is grounded in current physicochemical literature and serves as a practical starting point for researchers seeking to improve the translational value of in vitro assays.

## Figures and Tables

**Table 2:** Approximate intracellular concentrations of essential cations in mammalian cells.

Essential Cations	Approximate Intracellular Concentration	Source
Calcium (Ca)	10^−1^–10^−4^ mM	[105]
Cobalt (Co)	Low Concentration, dependent on cell exposure	[103]
Copper (Cu)	Zeptomolar to femtomolar	[93]
Iron (Fe)	1–7 μM	[102]
Magnesium (Mg)	17–20 mM total, (0.5–1 mM free)	[99]
Manganese (Mn)	low μΜ to sub-mM	[101]
Molybdenum (Mo)	5 nm, can vary	[104]
Nickel (Ni)	Very low: not defined concentration in mammals	[93]
Potassium (K)	140–150 mM	[98]
Sodium (Na)	10 mM	[95,96]
Zinc (Zn)	200–300 μΜ	[106]

**Table 3:** Concentrations of different ions in the PBS solution, not accounting for pH titration with NaOH or HCl.

Ion	Concentration in solution with pH 7.4, without CaCl_2_·2H_2_O and MgCl_2_·6H_2_O
Na^+^	157 mM
K^+^	4.50 mM
Cl^−^	139.70 mM
HPO_4_^2−^	7.23 mM
H_2_PO_4_^−^	4.57 mM

**Table 4:** Concentrations of different ions in the extracellular saline buffer and HHBS, not accounting for pH titration with NaOH or HCl.

Ion	Concentration in Extracellular Saline Buffer	Concentration in HHBS (pH 7.3, 25 °C)
Na^+^	140 mM	142.85 mM
K^+^	5.40 mM	5.77 mM
Cl^−^	149.40 mM	146.83 mM
Ca^2+^	2 mM	1.26 mM
Mg^2+^	10 mM	0.90 mM
SO_4_^2−^	10 mM	0.41 mM
HEPES	5 mM	20 mM
H_2_PO_4_^−^	-	0.44 mM
HPO_4_^2−^	-	0.34 mM
HCO_3_^−^	-	4.17 mM

**Table 5:** Suggested physicochemical parameters and components for the formulation of a cytoplasm-mimicking buffer.

Parameter	Suggested Value/Strategy	Notes
**Temperature**	37 °C	Must match physiological conditions for accurate thermodynamic constants
**pH**	~7.2–7.4 typical cytoplasmic 7.2 (nuclear studies); 7.1–7.7 in tumor cells	Should be tailored to target cell type
**Main Cations**	140–150 mM K^+^ 10 mM Na^+^ 1 mM Mg^2+^	K^+^ predominant over Na^+^ and Mg^2+^
**Main Anions**	95–120 mM Cl^−^ 12 mM HCO_3_^−^ or	NaHCO_3_^−^ may be used as a weak buffer
**Cosolvent for lipophilicity**	<3%	Although there is no literature to support certain values, some relevant studies include [28,41,140,141]. Certain proteins may exhibit sensitivity even at concentrations as low as 3% [141].
**Other Metal ions**	Zn^2+^, Ca^2+^, etc.	May be used if enzyme cofactors or structural ions are required
**Crowding Agents**	Peg 8000/Ficoll 70/Dextran (10–35%)	

## Data Availability

Data sharing is not applicable.

## Abbreviations

The following abbreviations are used in this manuscript:

ATP	Adenosine Triphosphate
Bc	Biochemical
BcA	Biochemical assay
BPS	Biochemical buffers
BS	Buffer solution
Ca	Calcium
CaCl_2_·2H_2_O	Calcium chloride dihydrate
Cb	Cell-based
CBA	Cell-based assays
CETSA	Cellular Thermal Shift Assay
Cl	Chloride
Co	Cobalt
CO_2_	Carbon dioxide
Cu	Copper
D(t)	Time-dependent diffusion coefficient
DTT	Dithiothreitol
dH_2_O	Distilled water
DMSO	Dimethyl sulfoxide
Fe	Ferrum
H_2_PO_4_^−^	Dihydrogen phosphate
HCO_3_^−^	Bicarbonate
HEPES	(4-(2-hydroxyethyl)-1-piperazineethanesulfonic acid)
HHBS	Hank’s Buffer with HEPES
HPO_4_^2−^	Hydrogen phosphate
IC_50_	Half-maximal inhibitory concentration
IDP	Intrinsically disordered protein
K	Potassium
K_a_	equilibrium association constant
KCl	Potassium chloride
K_d_	equilibrium dissociation constant
KH_2_PO_4_	Potassium dihydrogen phosphate
K_i_	Inhibition constant
K_m_	Michaelis-Menten constant
logD	Distribution coefficient
logP	Partition coefficient
MAPK	Mitogen-activated protein kinase
Mg	Magnesium
MgCl_2_·6H_2_O	Magnesium chloride hexahydrate
MgSO_4_	Magnesium sulfate
MgSO_4_·7H_2_O	Magnesium sulfate heptahydrate
Mn	Manganese
Mo	Molybdenum
M-PER	Mammalian protein extraction reagent
MSD	Mean Square Displacement
Na	Sodium
Na_2_HPO_4_	Disodium phosphate
NaCl	Sodium chloride
NaH_2_PO_4_	Sodium dihydrogen phosphate
NaHCO_3_	Sodium bicarbonate
NaOH	Sodium hydroxide
NHE	Na^+^/H^+^ exchangers
Ni	Nickel
NMR	Nuclear magnetic resonance
PBS	Phosphate-Buffered Saline
PCh	Physicochemical
PDB	Protein Data Bank
PEG	Polyethylene glycol
PGK	Phosphoglycerate kinase
pHe	Extracellular pH
pHi	Intracellular pH
Pi	Inorganic phosphate
S	Entropy
SAR	Structure Activity Relationship
SL2	Stem loop 2
SO_4_^−2^	Sulfate
T	Temperature
TBS	Tris-buffered saline
TNF	Tumor Necrosis Factor
TPSA	Topological polar surface area
V	Vanadium
VlsE	variable major protein-like sequence expressed
W	Tungsten
Zn	Zinc
ΔG	Change in Gibbs free energy
ΔH	Change in enthalpy
ΔS	Change in entropy

## References

[1] Pinne M., Raucy J.L. (2014). Advantages of cell-based high-volume screening assays to assess nuclear receptor activation during drug discovery. Expert Opin. Drug Discov..

[2] Guin D., Gruebele M. (2020). Chaperones Hsc70 and Hsp70 Bind to the Protein PGK Differently inside Living Cells. J. Phys. Chem. B.

[3] Wang S., Midgley C.A., Scaërou F., Grabarek J.B., Griffiths G., Jackson W., Kontopidis G., McClue S.J., McInnes C., Meades C. (2010). Discovery of N-phenyl-4-(thiazol-5-yl)pyrimidin-2-amine aurora kinase inhibitors. J. Med. Chem..

[4] Alexiou P., Papakyriakou A., Ntougkos E., Papaneophytou C.P., Liepouri F., Mettou A., Katsoulis I., Maranti A., Tsiliouka K., Strongilos A. (2014). Rationally designed less toxic SPD-304 analogs and preliminary evaluation of their TNF inhibitory effects. Arch. Pharm..

[5] Lipinski C.A., Lombardo F., Dominy B.W., Feeney P.J. (2001). Experimental and computational approaches to estimate solubility and permeability in drug discovery and development settings. Adv. Drug Deliv. Rev..

[6] Chiosis G., Huezo H., Rosen N., Mimnaugh E., Whitesell L., Neckers L. (2003). 17AAG: Low Target Binding Affinity and Potent Cell Activity—Finding an Explanation1. Mol. Cancer Ther..

[7] Knapinska A.M., Singh C., Drotleff G., Blanco D., Chai C., Schwab J., Herd A., Fields G.B. (2021). Matrix Metalloproteinase 13 Inhibitors for Modulation of Osteoclastogenesis: Enhancement of Solubility and Stability. ChemMedChem.

[8] Hong J.Y., Price I.R., Bai J.J., Lin H. (2019). A Glycoconjugated SIRT2 Inhibitor with Aqueous Solubility Allows Structure-Based Design of SIRT2 Inhibitors. ACS Chem. Biol..

[9] Mateus A., Gordon L.J., Wayne G.J., Almqvist H., Axelsson H., Seashore-Ludlow B., Treyer A., Matsson P., Lundbäck T., West A. (2017). Prediction of intracellular exposure bridges the gap between target-and cell-based drug discovery. Proc. Natl. Acad. Sci. USA.

[10] Lee E.J., Duggirala K.B., Lee Y., Yun M.R., Jang J., Cyriac R., Jung M.E., Choi G., Chae C.H., Cho B.C. (2022). Novel allosteric glutaminase 1 inhibitors with macrocyclic structure activity relationship analysis. Bioorg. Med. Chem. Lett..

[11] Luchinat E., Barbieri L., Cremonini M., Nocentini A., Supuran C.T., Banci L. (2020). Drug Screening in Human Cells by NMR Spectroscopy Allows the Early Assessment of Drug Potency. Angew. Chem. Int. Ed..

[12] Luzzio M.J., Besterman J.M., Emerson D.L., Evans M.G., Lackey K., Leitner P.L., McIntyre G., Morton B., Myers P.L., Peel M. (1995). Synthesis and antitumor activity of novel water soluble derivatives of camptothecin as specific inhibitors of topoisomerase I. J. Med. Chem..

[13] Kontopidis G., Andrews M.J.I., McInnes C., Cowan A., Powers H., Innes L., Plater A., Griffiths G., Paterson D., Zheleva D.I. (2003). Insights into Cyclin Groove Recognition: Complex Crystal Structures and Inhibitor Design through Ligand Exchange. Structure.

[14] Wu S.Y., McNae I., Kontopidis G., McClue S.J., McInnes C., Stewart K.J., Wang S., Zheleva D.I., Marriage H., Lane D.P. (2003). Discovery of a Novel Family of CDK Inhibitors with the Program LIDAEUS: Structural Basis for Ligand-Induced Disordering of the Activation Loop. Structure.

[15] Rinotas V., Liepouri F., Ouzouni M.-D., Chalkidi N., Papaneophytou C., Lampropoulou M., Vidali V.P., Kontopidis G., Couladouros E., Eliopoulos E. (2023). Structure-Based Discovery of Receptor Activator of Nuclear Factor-κB Ligand (RANKL)-Induced Osteoclastogenesis Inhibitors. Int. J. Mol. Sci..

[16] Camara R., Ogbeni D., Gerstmann L., Ostovar M., Hurer E., Scott M., Mahmoud N.G., Radon T., Crnogorac-Jurcevic T., Patel P. (2020). Discovery of novel small molecule inhibitors of S100P with in vitro anti-metastatic effects on pancreatic cancer cells. Eur. J. Med. Chem..

[17] Jung F.H., Pasquet G., Lambert-van der Brempt C., Lohmann J.-J.M., Warin N., Renaud F., Germain H., De Savi C., Roberts N., Johnson T. (2006). Discovery of Novel and Potent Thiazoloquinazolines as Selective Aurora A and B Kinase Inhibitors. J. Med. Chem..

[18] Hamada S., Suzuki T., Mino K., Koseki K., Oehme F., Flamme I., Ozasa H., Itoh Y., Ogasawara D., Komaarashi H. (2010). Design, Synthesis, Enzyme-Inhibitory Activity, and Effect on Human Cancer Cells of a Novel Series of Jumonji Domain-Containing Protein 2 Histone Demethylase Inhibitors. J. Med. Chem..

[19] Suzuki T., Nagano Y., Kouketsu A., Matsuura A., Maruyama S., Kurotaki M., Nakagawa H., Miyata N. (2005). Novel inhibitors of human histone deacetylases: Design, synthesis, enzyme inhibition, and cancer cell growth inhibition of SAHA-based non-hydroxamates. J. Med. Chem..

[20] Luby-Phelps K. (1999). Cytoarchitecture and physical properties of cytoplasm: Volume, viscosity, diffusion, intracellular surface area. Int. Rev. Cytol..

[21] Luby-Phelps K. (1994). Physical properties of cytoplasm. Curr. Opin. Cell Biol..

[22] Papaioannou O.S.E., Tsika A.C., Rovoli M., Papadopoulos G.E., Kontopidis G., Spyroulias G.A., Leonidas D.D. (2023). Structural and Biochemical Characterization of the Human Angiogenin–Proliferating Cell Nuclear Antigen Interaction. Biochemistry.

[23] Lázár L., Tsagkarakou A.S., Stravodimos G., Kontopidis G., Leffler H., Nilsson U.J., Somsák L., Leonidas D.D. (2023). Strong Binding of C-Glycosylic1, 2-Thiodisaccharides to Galectin-3—Enthalpy-Driven Affinity Enhancement by Water-Mediated Hydrogen Bonds. J. Med. Chem..

[24] Zhang A., Guo Z., Ge G., Liu Z. (2023). Insights into In Vivo Environmental Effects on Quantitative Biochemistry in Single Cells. Anal. Chem..

[25] Clegg J.S. (1984). Properties and metabolism of the aqueous cytoplasm and its boundaries. Am. J. Physiol. Integr. Comp. Physiol..

[26] Schnell S., Turner T.E. (2004). Reaction kinetics in intracellular environments with macromolecular crowding: Simulations and rate laws. Prog. Biophys. Mol. Biol..

[27] Stadmiller S.S., Aguilar J.S., Parnham S., Pielak G.J. (2020). Protein–Peptide Binding Energetics under Crowded Conditions. J. Phys. Chem. B.

[28] Papaneophytou C.P., Grigoroudis A.I., McInnes C., Kontopidis G. (2014). Quantification of the effects of ionic strength, viscosity, and hydrophobicity on protein-ligand binding affinity. ACS Med. Chem. Lett..

[29] Luh L.M., Hänsel R., Löhr F., Kirchner D.K., Krauskopf K., Pitzius S., Schäfer B., Tufar P., Corbeski I., Güntert P. (2013). Molecular crowding drives active Pin1 into nonspecific complexes with endogenous proteins prior to substrate recognition. J. Am. Chem. Soc..

[30] Seashore-Ludlow B., Axelsson H., Almqvist H., Dahlgren B., Jonsson M., Lundback T. (2018). Quantitative interpretation of intracellular drug binding and kinetics using the cellular thermal shift assay. Biochemistry.

[31] Chen Q., Schönherr H., Vancso G.J. (2009). Block-Copolymer Vesicles as Nanoreactors for Enzymatic Reactions. Small.

[32] Norris M.G.S., Malys N. (2011). What is the true enzyme kinetics in the biological system? An investigation of macromolecular crowding effect upon enzyme kinetics of glucose-6-phosphate dehydrogenase. Biochem. Biophys. Res. Commun..

[33] Fulton A.B. (1982). How crowded is the cytoplasm?. Cell.

[34] Golding I., Cox E.C. (2006). Physical Nature of Bacterial Cytoplasm. Phys. Rev. Lett..

[35] McGuffee S.R., Elcock A.H. (2010). Diffusion, crowding & protein stability in a dynamic molecular model of the bacterial cytoplasm. PLoS Comput. Biol..

[36] Rivas G., Minton A.P. (2018). Toward an understanding of biochemical equilibria within living cells. Biophys. Rev..

[37] Rivas G., Minton A.P. (2020). Editorial: Biochemical Reactions in Cytomimetic Media. Front. Mol. Biosci..

[38] Hann M.M., Simpson G.L. (2014). Intracellular drug concentration and disposition—The missing link?. Methods.

[39] Wu S.Y., Dornan J., Kontopidis G., Taylor P., Walkinshaw M.D. (2001). The First Direct Determination of a Ligand Binding Constant in Protein Crystals. Angew. Chem. Int. Ed. Engl..

[40] Mettou A., Papaneophytou C., Melagraki G., Maranti A., Liepouri F., Alexiou P., Papakyriakou A., Couladouros E., Eliopoulos E., Afantitis A. (2018). Aqueous Solubility Enhancement for Bioassays of Insoluble Inhibitors and QSPR Analysis: A TNF-α Study. SLAS Discov. Adv. Life Sci. RD.

[41] Papaneophytou C.P., Mettou A.K., Rinotas V., Douni E., Kontopidis G.A. (2013). Solvent Selection for Insoluble Ligands, a Challenge for Biological Assay Development: A TNF-α/SPD304 Study. ACS Med. Chem. Lett..

[42] Busa W.B., Nuccitelli R. (1984). Metabolic regulation via intracellular pH. Am. J. Physiol. Integr. Comp. Physiol..

[43] Marakhova I., Yurinskaya V., Aksenov N., Zenin V., Shatrova A., Vereninov A. (2019). Intracellular K+ and water content in human blood lymphocytes during transition from quiescence to proliferation. Sci. Rep..

[44] Morse P.D. (1986). Determining Intracellular Viscosity from the Rotational Motion of Spin Labels. Methods in Enzymology.

[45] Roos A., Boron W.F. (1981). Intracellular pH. Physiol. Rev..

[46] Tverskoi A.M., Sidorenko S.V., Klimanova E.A., Akimova O.A., Smolyaninova L.V., Lopina O.D., Orlov S.N. (2016). Effects of ouabain on proliferation of human endothelial cells correlate with Na+,K+-ATPase activity and intracellular ratio of Na+ and K+. Biochemistry.

[47] Banerjee R. (2012). Redox outside the box: Linking extracellular redox remodeling with intracellular redox metabolism. J. Biol. Chem..

[48] Kaludercic N., Deshwal S., Di Lisa F. (2014). Reactive oxygen species and redox compartmentalization. Front. Physiol..

[49] Go Y.M., Jones D.P. (2008). Redox compartmentalization in eukaryotic cells. Biochim. Biophys. Acta-Gen. Subj..

[50] Pajares M., Jiménez-Moreno N., Dias I.H.K., Debelec B., Vucetic M., Fladmark K.E., Basaga H., Ribaric S., Milisav I., Cuadrado A. (2015). Redox control of protein degradation. Redox Biol..

[51] Lee H., Torres J., Truong L., Chaudhuri R., Mittal A., Johnson M.E. (2012). Reducing agents affect inhibitory activities of compounds: Results from multiple drug targets. Anal. Biochem..

[52] Overington J.P., Al-Lazikani B., Hopkins A.L. (2006). How many drug targets are there?. Nat. Rev. Drug Discov..

[53] Gusenbauer M. (2019). Google Scholar to overshadow them all? Comparing the sizes of 12 academic search engines and bibliographic databases. Scientometrics.

[54] Luby-Phelps K. (2013). The physical chemistry of cytoplasm and its influence on cell function: An update. Mol. Biol. Cell.

[55] van der Vegt N.F.A., Nayar D. (2017). The Hydrophobic Effect and the Role of Cosolvents. J. Phys. Chem. B.

[56] Casey J.R., Grinstein S., Orlowski J. (2010). Sensors and regulators of intracellular pH. Nat. Rev. Mol. Cell Biol..

[57] Kneen M., Farinas J., Li Y., Verkman A.S. (1998). Green Fluorescent Protein as a Noninvasive Intracellular pH Indicator. Biophys. J..

[58] Han J., Burgess K. (2010). Fluorescent Indicators for Intracellular pH. Chem. Rev..

[59] Onufriev A.V., Alexov E. (2013). Protonation and pK changes in protein-ligand binding. Q. Rev. Biophys..

[60] Gillies R.J., Raghunand N., Garcia-Martin M.L., Gatenby R.A. (2004). pH imaging. IEEE Eng. Med. Biol. Mag..

[61] Rolver M.G., Pedersen S.F. (2021). Putting Warburg to work: How imaging of tumour acidosis could help predict metastatic potential in breast cancer. Br. J. Cancer.

[62] Doyen D., Poet M., Jarretou G., Pisani D.F., Tauc M., Cougnon M., Argentina M., Bouret Y., Counillon L. (2022). Intracellular pH Control by Membrane Transport in Mammalian Cells. Insights Into the Selective Advantages of Functional Redundancy. Front. Mol. Biosci..

[63] Boedtkjer E., Pedersen S.F. (2020). The Acidic Tumor Microenvironment as a Driver of Cancer. Annu. Rev. Physiol..

[64] Reshkin S.J., Greco M.R., Cardone R.A. (2014). Role of pHi, and proton transporters in oncogene-driven neoplastic transformation. Philos. Trans. R. Soc. Biol. Sci..

[65] White K.A., Grillo-Hill B.K., Barber D.L. (2017). Cancer cell behaviors mediated by dysregulated pH dynamics at a glance. J. Cell Sci..

[66] Segeritz C.-P., Vallier L. (2017). Cell Culture. Basic Science Methods for Clinical Researchers.

[67] Sherman T.A., Rongali S.C., Matthews T.A., Pfeiffer J., Nehrke K. (2012). Identification of a nuclear carbonic anhydrase in Caenorhabditis elegans. Biochim. Biophys. Acta BBA-Mol. Cell Res..

[68] Yong M.J., Kang B., Yang U., Oh S.S., Je J.H. (2022). Live Streaming of a Single Cell’s Life over a Local pH-Monitoring Nanowire Waveguide. Nano Lett..

[69] Hou H., Zhao Y., Li C., Wang M., Xu X., Jin Y. (2017). Single-cell pH imaging and detection for pH profiling and label-free rapid identification of cancer-cells. Sci. Rep..

[70] Ganji M., Docter M., Le Grice S.F.J., Abbondanzieri E.A. (2016). DNA binding proteins explore multiple local configurations during docking via rapid rebinding. Nucleic Acids Res..

[71] Feig M. (2021). Virtual Issue on Protein Crowding and Stability. J. Phys. Chem. B.

[72] Neurohr G.E., Amon A. (2020). Relevance and Regulation of Cell Density. Trends Cell Biol..

[73] Molines A.T., Lemiere J., Gazzola M., Steinmark I.E., Edrington C.H., Hsu C.T., Real-Calderon P., Suhling K., Goshima G., Holt L.J. (2022). Physical properties of the cytoplasm modulate the rates of microtubule polymerization and depolymerization. Dev. Cell.

[74] Nenninger A., Mastroianni G., Mullineaux C.W. (2010). Size dependence of protein diffusion in the cytoplasm of Escherichia coli. J. Bacteriol..

[75] Haspinger D.C., Klinge S., Holzapfel G.A. (2021). Numerical analysis of the impact of cytoskeletal actin filament density alterations onto the diffusive vesicle-mediated cell transport. PLoS Comput. Biol..

[76] Potma E.O., De Boeij W.P., Bosgraaf L., Roelofs J., Van Haastert P.J.M., Wiersma D.A. (2001). Reduced protein diffusion rate by cytoskeleton in vegetative and polarized Dictyostelium cells. Biophys. J..

[77] Pei D., Buyanova M. (2017). Overcoming Endosomal Entrapment in Drug Delivery. Bioconjugate Chem..

[78] Zhitomirsky B., Assaraf Y.G. (2017). Lysosomal accumulation of anticancer drugs triggers lysosomal exocytosis. Oncotarget.

[79] Lismont C., Nordgren M., Van Veldhoven P.P., Fransen M. (2015). Redox interplay between mitochondria and peroxisomes. Front. Cell Dev. Biol..

[80] Mazurkiewicz J., Nowotny-Różańska M. (1998). Viscosity of aqueous solutions of saccharides. Polish J. Food Nutr. Sci..

[81] Lavrenko P.N., Mikryukova O.I., Didenko S.A. (1986). Hydrodynamic properties and the shape of the molecules of the polysaccharide ficoll-400 in solution. Polym. Sci. USSR.

[82] Ernst D., Hellmann M., Köhler J., Weiss M. (2012). Fractional Brownian motion in crowded fluids. Soft Matter.

[83] El-Morched M., Barraco C., Simionescu R., Harroun T., Yan H. (2024). Characterization of Simulated Cytoplasmic Fluids. ChemistrySelect.

[84] Chalikian T.V. (2016). Excluded volume contribution to cosolvent-mediated modulation of macromolecular folding and binding reactions. Biophys. Chem..

[85] Dupuis N.F., Holmstrom E.D., Nesbitt D.J. (2014). Molecular-crowding effects on single-molecule RNA folding/unfolding thermodynamics and kinetics. Proc. Natl. Acad. Sci. USA.

[86] Rajendran D., Mitra S., Oikawa H., Madhurima K., Sekhar A., Takahashi S., Naganathan A.N. (2022). Quantification of Entropic Excluded Volume Effects Driving Crowding-Induced Collapse and Folding of a Disordered Protein. J. Phys. Chem. Lett..

[87] Roque A., Ponte I., Suau P. (2007). Macromolecular crowding induces a molten globule state in the C-terminal domain of histone H1. Biophys. J..

[88] Altamash T., Ahmed W., Rasool S., Biswas K.H. (2021). Intracellular Ionic Strength Sensing Using NanoLuc. Int. J. Mol. Sci..

[89] Jomova K., Makova M., Alomar S.Y., Alwasel S.H., Nepovimova E., Kuca K., Rhodes C.J., Valko M. (2022). Essential metals in health and disease. Chem.-Biol. Interact..

[90] Peters K., Staehlke S., Rebl H., Jonitz-Heincke A., Hahn O. (2024). Impact of Metal Ions on Cellular Functions: A Focus on Mesenchymal Stem/Stromal Cell Differentiation. Int. J. Mol. Sci..

[91] Nielsen F. (2021). Nickel. Adv. Nutr..

[92] Brooke D., Movahed N., Bothner B. (2015). Universal buffers for use in biochemistry and biophysical experiments. AIMS Biophys..

[93] Patterson C.J., Pernil R., Foster A.W., Robinson N.J., Culotta V., Scott R.A. (2013). Cyanobacterial Models that Address Cross-Talk in Metal Homeostasis. Metals in Cells.

[94] Permyakov E.A. (2021). Metal Binding Proteins. Encyclopedia.

[95] Lodish H., Kaiser C.A., Krieger M., Bretscher A., Ploegh H., Martin K.C., Yaffe M., Amon A. (2021). Transmembrane Transport of Ions and Small Molecules. Molecular Cell Biology.

[96] Strazzullo P., Leclercq C. (2014). Sodium. Adv. Nutr..

[97] Burkitt Creedon J.M. (2015). Sodium Disorders. Small Animal Critical Care Medicine.

[98] Zacchia M., Abategiovanni M.L., Stratigis S., Capasso G. (2016). Potassium: From Physiology to Clinical Implications. Kidney Dis..

[99] Romani A.M.P., Vink R N.M. (2011). Intracellular*magnesium*homeostasis. Magnesium in the Central Nervous System.

[100] Ramappa V.K., Srivastava D., Singh P., Kumar U., Kumar D., Gosipatala S.B., Saha S., Kumar D., Raj R. (2020). Mulberries: A Promising Fruit for Phytochemicals, Nutraceuticals, and Biological Activities. Int. J. Fruit Sci..

[101] Das S., Khatua K., Rakshit A., Carmona A., Sarkar A., Bakthavatsalam S., Ortega R., Datta A. (2019). Emerging chemical tools and techniques for tracking biological manganese. Dalt. Trans..

[102] Philpott C.C., Jadhav S. (2019). The ins and outs of iron: Escorting iron through the mammalian cytosol. Free. Radic. Biol. Med..

[103] Simonsen L.O., Harbak H., Bennekou P. (2012). Cobalt metabolism and toxicology—A brief update. Sci. Total Environ..

[104] Institute of Medicine (US) Panel on Micronutrients (2001). Molybdenum. Dietary Reference Intakes for Vitamin A, Vitamin K, Arsenic, Boron, Chromium, Copper, Iodine, Iron, Manganese, Molybdenum, Nickel, Silicon, Vanadium, and Zinc.

[105] Lodish H., Berk A., Kaiser C.A., Krieger M., Bretscher A., Ploegh H., Martin K.C., Yaffe M., Amon A. (2021). Culturing and Visualizing Cells. Molecular Cell Biology.

[106] Chen B., Yu P., Chan W.N., Xie F., Zhang Y., Liang L., Leung K.T., Lo K.W., Yu J., Tse G.M.K. (2024). Cellular zinc metabolism and zinc signaling: From biological functions to diseases and therapeutic targets. Signal Transduct. Target. Ther..

[107] Kadhim M.J., Gamaj M.I. (2020). Estimation of the Diffusion Coefficient and Hydrodynamic Radius (stokes Radius) for Inorganic Ions in Solution Depending on Molar Conductivity as Electro-Analytical Technique—A Review. J. Chem. Rev..

[108] Kritmetapak K., Kumar R. (2021). Phosphate as a Signaling Molecule. Calcif. Tissue Int..

[109] Shcheynikov N., Son A., Hong J.H., Yamazaki O., Ohana E., Kurtz I., Shin D.M., Muallem S. (2015). Intracellular Cl- as a signaling ion that potently regulates Na^+^/HCO3^−^ transporters. Proc. Natl. Acad. Sci. USA.

[110] Koulouridis I., Koulouridis E. (2023). The Integral Role of Chloride & With-No-Lysine Kinases in Cell Volume Regulation & Hypertension. Int. J. Nephrol. Renov. Dis..

[111] Markovich D. (2001). Physiological Roles and Regulation of Mammalian Sulfate Transporters. Physiol. Rev..

[112] Kucernak A.R., Wang H., Lin X. (2024). Avoid Using Phosphate Buffered Saline (PBS) as an Electrolyte for Accurate OER Studies. ACS Energy Lett..

[113] AAT Bioquest Inc (2025). Phosphate Buffer (pH 5.8 to 7.4) Preparation and Recipe. https://www.aatbio.com/resources/buffer-preparations-and-recipes/phosphate-buffer-ph-5-8-to-7-4.

[114] National Center for Biotechnology Information (2025). PubChem Compound Summary for CID 23831, Hepes. https://pubchem.ncbi.nlm.nih.gov/compound/Hepes.

[115] AAT Bioquest Inc (2025). HHBS (Hank’s Buffer with Hepes) Preparation and Recipe. https://www.aatbio.com/resources/buffer-preparations-and-recipes/hhbs-hanks-buffer-with-hepes.

[116] AAT Bioquest Inc (2025). HEPES Buffer (1 M, 7.5 pH) Preparation and Recipe. https://www.aatbio.com/resources/buffer-preparations-and-recipes/hepes-buffer-ph-7-5.

[117] Green M.R., Sambrook J. (2018). Buffers. Cold Spring Harb Protoc..

[118] National Center for Biotechnology Information PubChem Compound Summary for CID 6503, Tris(hydroxymethyl)aminomethane. https://pubchem.ncbi.nlm.nih.gov/compound/Tris_hydroxymethyl_aminomethane.

[119] AAT Bioquest Inc (2025). Tris Buffer (1 M, pH 7.2) Preparation and Recipe. https://www.aatbio.com/resources/buffer-preparations-and-recipes/tris-buffer.

[120] AAT Bioquest Inc (2025). Tris-Buffered Saline (TBS, 0.1 M) Preparation and Recipe. https://www.aatbio.com/resources/buffer-preparations-and-recipes/tris-buffered-saline-tbs-0-1-m.

[121] Chmiel T., Mieszkowska A., Kempińska-Kupczyk D., Kot-Wasik A., Namieśnik J., Mazerska Z. (2019). The impact of lipophilicity on environmental processes, drug delivery and bioavailability of food components. Microchem. J..

[122] Liu X., Testa B., Fahr A. (2011). Lipophilicity and its relationship with passive drug permeation. Pharm. Res..

[123] Reckitt Benckiser (2012). Partition and distribution coefficients. RSC Adv. Chem. Sci..

[124] Wu K., Kwon S.H., Zhou X., Fuller C., Wang X., Vadgama J., Wu Y. (2024). Overcoming Challenges in Small-Molecule Drug Bioavailability: A Review of Key Factors and Approaches. Int. J. Mol. Sci..

[125] Asokan A., Cho M.J. (2002). Exploitation of Intracellular pH Gradients in the Cellular Delivery of Macromolecules. J. Pharm. Sci..

[126] Davis C.M., Gruebele M. (2021). Cellular Sticking Can Strongly Reduce Complex Binding by Speeding Dissociation. J. Phys. Chem. B.

[127] Davis C.M., Deutsch J., Gruebele M. (2020). An in vitro mimic of in-cell solvation for protein folding studies. Protein Sci..

[128] Knab E., Davis C.M. (2023). Chemical interactions modulate λ6-85 stability in cells. Protein Sci..

[129] Yoo H., Davis C.M. (2022). An in Vitro Cytomimetic of In-Cell RNA Folding. ChemBioChem.

[130] Luchinat E., Banci L. (2022). In-cell NMR: From target structure and dynamics to drug screening. Curr. Opin. Struct. Biol..

[131] Molina D.M., Jafari R., Ignatushchenko M., Seki T., Larsson E.A., Dan C., Sreekumar L., Cao Y., Nordlund P. (2013). Monitoring drug target engagement in cells and tissues using the cellular thermal shift assay. Science.

[132] Jafari R., Almqvist H., Axelsson H., Ignatushchenko M., Lundbäck T., Nordlund P., Molina D.M. (2014). The cellular thermal shift assay for evaluating drug target interactions in cells. Nat. Protoc..

[133] Mansouritorghabeh H., Jabbari-Azad F., Varasteh A., Sankian M., Farid-Hosseini R. (2017). Common solvents for making extraction of allergenic proteins from plants’ pollens for prick tests and related factors: A technical review. Electron. Physician.

[134] Yu B., Pettitt B.M., Iwahara J. (2020). Dynamics of Ionic Interactions at Protein-Nucleic Acid Interfaces. Acc. Chem. Res..

[135] Good N.E., Winget G.D., Winter W., Connolly T.N., Izawa S., Singh R.M.M. (1966). Hydrogen Ion Buffers for Biological Research. Biochemistry.

[136] Michl J., Park K.C., Swietach P. (2019). Evidence-based guidelines for controlling pH in mammalian live-cell culture systems. Commun. Biol..

[137] Delmouly K., Belondrade M., Casanova D., Milhavet O., Lehmann S. (2011). HEPES inhibits the conversion of prion protein in cell culture. J. Gen. Virol..

[138] Padan E., Schuldiner S. (1986). Intracellular pH regulation in bacterial cells. Methods in Enzymology.

[139] Tritsch G.L., Niswander P.R., Rosenfeld J., Nechaev A., Mittelman A. (1976). Cosolvent-buffer mixtures as models for the cytoplasmic milieu: The enzymology of adenosine aminohydrolase. Mol. Cell. Biochem..

[140] Wallerstein J., Akke M. (2019). Minute Additions of DMSO Affect Protein Dynamics Measurements by NMR Relaxation Experiments through Significant Changes in Solvent Viscosity. ChemPhysChem.

[141] Chan D.S.H., Kavanagh M.E., McLean K.J., Munro A.W., Matak-Vinković D., Coyne A.G., Abell C. (2017). Effect of DMSO on Protein Structure and Interactions Assessed by Collision-Induced Dissociation and Unfolding. Anal. Chem..

[142] McNae I.W., Kan D., Kontopidis G., Patterson A., Taylor P., Worrall L., Walkinshaw M.D. (2005). Studying protein–ligand interactions using protein crystallography. Crystallogr. Rev..

[143] Lowe E.D., Tews I., Cheng K.Y., Brown N.R., Gul S., Noble M.E.M., Gamblin S.J., Johnson L.N. (2002). Specificity Determinants of Recruitment Peptides Bound to Phospho-CDK2/Cyclin A. Biochemistry.

[144] Keleti T. (1986). Two rules of enzyme kinetics for reversible Michaelis-Menten mechanisms. FEBS Lett..

[145] Ohno A., Inomata K., Tochio H., Shirakawa M. (2011). In-Cell NMR Spectroscopy in Protein Chemistry and Drug Discovery. Curr. Top. Med. Chem..

[146] Peters J., Oliva R., Caliò A., Oger P., Winter R. (2023). Effects of Crowding and Cosolutes on Biomolecular Function at Extreme Environmental Conditions. Chem. Rev..

[147] Bull S.C., Doig A.J. (2015). Properties of protein drug target classes. PLoS ONE.

